# HPV16/18 genotyping for the triage of HPV positive women in primary cervical cancer screening in Chile

**DOI:** 10.1186/s13027-015-0038-5

**Published:** 2015-11-23

**Authors:** Marcela Lagos, Vanessa Van De Wyngard, Helena Poggi, Paz Cook, Paola Viviani, María Isabel Barriga, Martha Pruyas, Catterina Ferreccio

**Affiliations:** Departamento de Laboratorios Clínicos, Facultad de Medicina, Pontificia Universidad Católica de Chile, Av. Vicuña Mackenna 4686, Macul, Santiago, 7820436 Chile; Departamento de Salud Pública, Escuela/Facultad de Medicina, Pontificia Universidad Católica de Chile, Marcoleta 434, Santiago, 8330073 Chile; Departamento de Obstetricia y Ginecología, Facultad de Medicina, Pontificia Universidad Católica de Chile, Lira 85, Santiago, 8330074 Chile; Departamento de Anatomía Patológica, Hospital Dr Sótero del Río, Av. Concha y Toro 3459, Puente Alto, Santiago, 8207257 Chile; Advanced Center for Chronic Diseases (ACCDis), Sergio Livingstone 1007, Independencia, Santiago, 8380492 Chile

**Keywords:** Cervical cancer screening, Human papillomavirus, HPV DNA testing, HPV 16, HPV 18, Genotype, Triage

## Abstract

**Background:**

We previously conducted a population-based screening trial of high-risk human papillomavirus (hrHPV) testing and conventional cytology, demonstrating higher sensitivity (92.7 % vs 22.1 % for CIN2+) but lower positive predictive value (10.5 % vs 23.9 %) of hrHPV testing. Here we report the performance of HPV16/18 genotyping to triage the hrHPV positive participants.

**Methods:**

Women aged 25 years and older received hrHPV (Hybrid Capture 2) and Papanicolaou testing; positives by either test underwent colposcopy and directed biopsy, as did a sample of double-negatives. hrHPV positive women were reflex-tested with HPV16/18 genotyping (Digene HPV Genotyping PS Test).

**Results:**

Among the 8,265 participants, 10.7 % were hrHPV positive, 1.7 % had ASCUS+ cytology, 1.2 % had CIN2+; 776 (88 %) hrHPV positive women had complete results, of whom 38.8 % were positive for HPV16 (24.0 %), HPV18 (9.7 %) or both (5.1 %). CIN2+ prevalence in HPV16/18 positive women (16.3 %, 95 % CI 12.3-20.9) was twice that of HPV16/18 negative women (8.0 %, 95 % CI 5.7-10.8). HPV16/18 genotyping identified 40.5 % of CIN2, 66.7 % of CIN3 and 75.0 % of cancers. Compared to hrHPV screening alone, HPV16/18 triage significantly reduced the referral rate (10.7 % vs 3.7 %) and the number of colposcopies required to detect one CIN2+ (9 vs 6). When HPV16/18 negative women with baseline ASCUS+ cytology were also colposcopied, an additional 14 % of CIN2+ was identified; referral increased slightly to 4.2 %.

**Conclusions:**

HPV16/18 triage effectively stratified hrHPV positive women by their risk of high-grade lesions. HPV16/18 positive women must be referred immediately; referral could be deferred in HPV16/18 negative women given the slower progression of non-HPV16/18 lesions, however, they will require active follow-up.

## Background

Chile has a well-organized nationwide cervical cancer prevention program which is based on Papanicolaou (Pap) testing every three years for women aged 25–64, with coverage around 60 % [1]. Despite this, Chile’s cervical cancer mortality remains high (adjusted rate of 5.8/100,000 in 2010) [2] and has a markedly unequal socio-economic distribution [3]. Therefore, it has become necessary to assess the implementation in our national program of the newly available prevention strategies based on human papillomavirus (HPV), the causative agent of cervical cancer.

High-risk HPV (hrHPV) testing is more sensitive in detecting high-grade cervical lesions [4], providing greater protection against invasive cervical carcinomas than cytology [5]. We previously studied the performance of Pap cytology and hrHPV DNA detection in the routine clinical practice of our public health centers, in more than 8,000 women from the general population of Santiago [3]. We found that HPV testing was four times more sensitive than Pap testing (92.7 % vs 22.1 %) for the detection of cervical intraepithelial neoplasia grade 2 or worse (CIN2+); however, it was less specific and its positive predictive value was half that of the Pap test (10.5 % vs 23.9 %). Using HPV testing as a stand-alone primary screening test would require referring ten times more women for colposcopy; since 90 % of the referred women would not have lesions, this increased referral would unnecessarily overload the public health system and possibly lead to over-diagnosis and over-treatment. Therefore, HPV-based primary screening requires the identification of an adequate triage method for hrHPV positive women, so as to further stratify them by their risk of having high-grade cervical lesions.

HPV16/18 genotyping is one of the triage methods being considered worldwide. Because HPV16 and HPV18 are associated with about 70 % of invasive cervical cancers [6, 7], their identification in cervical samples indicates an elevated risk of presenting high-grade lesions compared with only identifying other HR-HPVs. In the US, the 10-year cumulative risk of CIN3+ associated with one-time detection of HPVs 16 or 18 was five times higher than for other high-risk types (17 % and 14 % vs 3 %) [8]. In the Netherlands, the 18-month cumulative risk of CIN3+ was higher in women positive for HPVs 16 or 18 than those positive for other high-risk types, irrespective of their cytology results (13 % vs 2 % in women with normal cytology, and 67 % vs 51 % in women with high-grade cytology) [9]. In Denmark, the 12-year absolute risk of CIN3+ was higher for HPV16 persistence than for the persistence of any other high-risk type (47.4 % for HPV16 alone vs 19.3 % for the pool of 13 hrHPVs) [10].

Here we report the results of HPV16/18 reflex-testing in 776 hrHPV positive women from our screening trial. We aimed to assess the performance of this test as a triage method when hrHPV testing is the sole primary screening test.

## Results

**Fig. 1:**
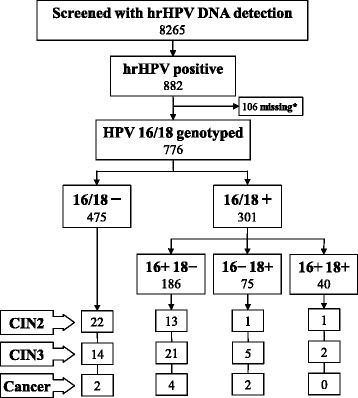
HPV16/18 genotyping results and outcomes of the hrHPV positive participants of a population-based screening trial in Chile, 2010. *Missing = 53 women with insufficient sample material for reflex testing plus 53 women who did not comply with colposcopy. hrHPV: high-risk human papillomavirus; CIN2+: cervical intraepithelial neoplasia grade 2 or worse; CIN3+: cervical intraepithelial neoplasia grade 3 or worse

From August 2009 to June 2010, 8,309 eligible and consenting women were enrolled in our screening trial, of whom 99.5 % had complete screening results: hrHPV positivity was 10.7 %, Pap test positivity at ASCUS or worse (ASCUS+) was 1.7 %, and CIN2+ prevalence was 1.2 %. The targets of the present study were the 882 hrHPV positive women. Genotyping results were available for 829 women (94.0 %), of whom 776 (93.6 %) had complied with colposcopy; compliance was equal in HPV16/18 positive and negative women (93.2 % and 93.9 %). Among the 776 women with complete results, HPV16/18 positivity was 38.8 %: 24.0 % were positive for HPV16 alone, 9.7 % for HPV18 alone, and 5.1 % for both genotypes (Fig. 1). CIN2+ prevalence was 11.2 %: 8.0 % (95 % CI 5.7-10.8) in HPV16/18 negative women and 16.3 % (12.3-20.9) in HPV16/18 positive women.

Among women with CIN2+ lesions, 56.3 % were HPV16/18 positive: 47.1 % were positive for HPV16 and 12.6 % for HPV18. HPV16/18 positivity increased with increasing lesion severity, from 40.5 % in women with CIN2 to 75.0 % in those with cancer. Table 1 shows the performance characteristics of HPV16/18 genotyping for the detection of high-grade cervical lesions among the hrHPV positive women; genotyping was less sensitive but more specific in women aged 30 years and older. Compared to hrHPV screening alone, HPV16/18 triage reduced the overall colposcopy referral rate from 10.7 % to 3.7 % (65 % reduction) and the number of colposcopies required to identify one lesion from 9.2 to 6.1 for CIN2+ (34 % reduction) and from 16.0 to 8.9 for CIN3+ (44 % reduction).

**Table 1:** Age-stratified performance of HPV16/18 genotyping for the detection of high-grade cervical lesions among 776 hrHPV positive women, Chile 2010.

			Sensitivity	Specificity	PPV	NPV
Lesion	Age	[n lesions]	% (95 % CI)			
CIN2+	<30	[24]	62.5	54.7	16.1	91.3
			(40.6–81.2)	(46.9–62.2)	(9.3–25.2)	(84.1–95.9)
	≥30	[63]	54.0	66.3	16.4	92.2
			(40.9–66.6)	(62.1–70.4)	(11.6–22.1)	(89.0–94.7)
	All	[87]	56.3	63.4	16.3	92.0
			(45.9–66.7)	(59.8–67.0)	(12.1–20.4)	(89.6–94.4)
CIN3+	<30	[11]	81.8	54.6	9.7	98.1
			(48.2–97.7)	(47.1–61.9)	(4.5–17.6)	(93.2–99.8)
	≥30	[39]	64.1	66.2	12.0	96.2
			(47.2–78.8)	(62.0–70.1)	(7.9–17.2)	(93.7–97.9)
	All	[50]	68.0	63.2	11.3	96.6
			(55.1–80.9)	(59.7–66.7)	(7.7–14.9)	(95.0–98.3)

*CIN2+* cervical intraepithelial neoplasia grade 2 or worse; *CIN3+* cervical intraepithelial neoplasia grade 3 or worse; *PPV* positive predictive value; *NPV* negative predictive value

An HPV screening strategy with HPV16/18 genotyping triage plus reflex cytology was explored. When baseline Pap test results (ASCUS cut-off) were considered to refer HPV16/18 negative women for colposcopy, 43 extra women were referred and 12 out of the 38 (31.6 %) women with CIN2+ lesions in this group were identified; the overall sensitivity of this strategy was 70 % with a total referral rate of 4.2 %. Among hrHPV positive women who were negative to HPV16/18, the risk of CIN2+ and CIN3+ was 27.9 % (12/43) and 18.6 % (8/43) for those with an abnormal Pap test, and 6.0 % (26/432) and 1.9 % (8/432) for those with a normal Pap test; therefore, the probability of having CIN2+ and CIN3+ lesions was 4.7 and 9.8 times higher in women with ASCUS+ cytology.

## Discussion

This study demonstrates that, in the context of a middle-developed Latin American country, HPV16/18 genotyping stratifies hrHPV positive women by their risk of presenting high-grade cervical lesions; HPV16/18 positive women had twice the risk of CIN2+ lesions than women only positive for any of 11 other hrHPV types. Although the latter women are still at moderate risk of CIN2+, precancerous lesions associated with hrHPVs other than 16, 18 and 45 are believed less likely to progress to cancer than those associated with the mentioned types [8–12]; this could indicate that most of the CIN2/3 lesions missed by HPV16/18 triage would be of lower risk, allowing a broader time window for referral. Still, 2 out of the 8 cancers identified in our study group were HPV16/18 negative, thus hrHPV positive HPV16/18 negative women must receive follow-up management.

Management options for HPV16/18 negative women include reflex cytology. In our study, additionally referring women with baseline ASCUS+ cytology considerably improved triage sensitivity with only a small increase in referrals. The gain in sensitivity obtained when adding cytology could be further increased if the performance of the cytological examination were improved by informing the reader of the HPV status of the sample, thus heightening awareness of the elevated risk of lesions [13].

Another alternative to manage HPV16/18 negative women is repeat HPV testing some time after the first positive test in order to identify hrHPV persistence. Deferring colposcopy for HPV16/18 negative women implies that some high-grade lesions will be missed at baseline and could progress. Therefore the key is to determine the interval before repeat testing that adequately balances minimizing the risk of progression and allowing enough time for clearance of transient infections. We re-examined 80 % of the HPV16/18 negative women in our study who did not have high-grade lesions at baseline: 47 % of them were retested (HC2) at 12–18 months, 26 % at 18–24 months and 27 % at 24–30 months; the corresponding clearance rates were 69 %, 57 % and 67 %. At the 12–18 month visit, more than 60 % of the women were already hrHPV negative and there was no further clearance beyond that time, thus this seems to be the ideal follow-up period in this population.

The performance of HPV16/18 genotyping to triage hrHPV positive women in our hands was comparable to those reported elsewhere. Among women with CIN2+ lesions, the prevalence of HPV16 in our study was similar to that reported in the ATHENA study (47.1 % vs 42.3 %), which used the cobas HPV test, while the prevalence of HPV18 was double that in ATHENA (12.6 % vs 6.2 %) [14]; our relatively high HPV18 prevalence is in line with that reported for HPV type-specific prevalence in Chilean women with high-grade lesions (HPV16: 55.5 % and HPV18: 15.5 %) [15]. We obtained a slightly higher sensitivity for HPV16/18 genotyping than that in the ATHENA study: 56.3 % vs 51.8 % for CIN2+ and 68.0 % vs 59.5 % for CIN3+ [16]. In the VUSA-Screen study in The Netherlands, which used HC2 and reverse line blot genotyping, the sensitivity of HPV16/18 genotyping for cumulatively detected CIN2+ over a 2-year period was 58.6 % [17]. In Sweden, the Swedescreen study used GP5+/6+ PCR enzyme immunoassay with reverse dot blot hibridization genotyping; the sensitivity of HPV16/18 genotyping for prevalent CIN2+ was 53.0 % [18].

There are other options to triage hrHPV positive women. In countries with established cytology programs such as Chile, reflex cytology seems particularly attractive. However, the very low sensitivity achieved by Pap testing in primary screening in our original study indicates that, as a triage test, it would nullify the high sensitivity of the HPV test. We were limited in our ability to assess the true performance of cytology for triage, given the design of our primary trial which required cytotechnicians to be unaware of HPV results. If the baseline Pap test were used to triage the hrHPV positive women in our study, 64 % of the CIN2+ lesions identified in these women would have been missed (compared to 44 % missed by HPV16/18 genotyping). Therefore, this would not be a safe alternative in Chile until quality of cytology as a triage test can be improved, achievable in part by prior knowledge of HPV status [13]. Cytology triage obtained higher sensitivity (53 % for CIN2+) in the ATHENA trial [16], and was even more sensitive (63 % for CIN2+) in the VUSA-Screen study [17]. This shows the large variation of cytology performance among different laboratories; thus, the implementation of cytology triage requires a stringent quality control system.

A limitation of our study is that we did not employ a pathology review panel, which could have affected our results to some degree due to misclassification of histology; since the pathologists were blinded to the HPV status of the samples, we do not expect systematic bias. Another possible limitation is that the samples were stored frozen for up to two years before genotyping. Long-term storage could lead to DNA degradation; however, it has been shown that HC2 testing is reproducible over time in frozen specimens [19] and it is likely that the same may occur with the PS test which is based on hybrid capture technology.

Our study is strengthened by its population-based design, large sample size, high response rate, and the fact that it was carried out within the setting of the Chilean national cervical cancer screening program; therefore it is likely that our results reflect those obtainable in the routine practice of our public health system.

## Conclusions

HPV16/18 genotyping was effective in stratifying hrHPV positive women into those with higher and lower risk of prevalent high-grade lesions, significantly reducing colposcopy referrals. hrHPV testing with HPV16/18 triage could be considered as a screening strategy to determine immediate referral. However, since triage-negative women remain at moderate risk, they must receive active follow-up management; the most adequate strategies in the context of each health system should be determined. Studies are underway to identify alternative triage methods that could provide higher sensitivity without compromising specificity.

## Methods

This study was approved by the ethics committees of the Pontificia Universidad Católica de Chile and the Servicio de Salud Metropilitano Sur-Oriente. All participants gave written informed consent before entering the study. The detailed methods have been described elsewhere [3]. Briefly, the study was conducted in a low to middle socio-economic area of Santiago, Chile, within the setting of the national cervical cancer program. Three public primary health centers and their referral hospital participated in the study with their regular infrastructure, personnel and protocols; only HPV testing was newly implemented. We invited women from the general population aged 25–64 who resided locally and were not pregnant, hysterectomized or virgins. This population had not received HPV vaccination (the HPV vaccine was incorporated into the public immunization program in 2014, targeting girls age 9–10 years). A total of 8,265 women received both hrHPV DNA detection and conventional Pap cytology; the 930 women who tested positive by either test underwent colposcopy and directed biopsy, as did a control sample of 295 screen-negative women. The current analysis is focused on the 776 hrHPV positive women with complete follow-up data (88 %) who were reflex tested for the presence of hrHPV 16 and 18 genotypes.

### hrHPV DNA testing and HPV16/18 genotyping

Both tests were performed at the Molecular Biology Laboratory of the Pontificia Universidad Católica de Chile University. Each sample was divided into two 500 ul aliquots. One was tested for hrHPV using the Hybrid Capture 2 High Risk HPV DNA test (HC2, Qiagen, Gaithersburg, USA), which detects viral DNA by nucleic acid hybridization and uses a pooled probe set for 13 oncogenic HPV genotypes (HPV 16, 18, 31, 33, 35, 39, 45, 51, 52, 56, 58, 59, 68); samples were considered positive when the relative light unit/cutoff was ≥1.0. The second aliquot was stored at −80° Celsius until genotyping, 1–2 years after collection. Genotyping was performed as a reflex test in HPV-positive women, using the Digene HPV Genotyping PS test (Qiagen, Gaithersburg, USA), currently available in Europe and Australia. This test is based on hybrid capture technology with a working protocol similar to the HC2 test, but in which each sample is tested against separate sets of type-specific probes allowing the individual detection of HPV16, HPV18 and HPV45 [20]; we only tested for HPV16 and HPV18. Reference cut-off values were determined per manufacturer’s instructions; samples were considered positive when the relative light unit/cutoff was ≥1.3.

### Conventional cytology

Cytologic evaluation was conducted at the area referral hospital, following regular protocols, with results reported according to the 2011 Bethesda System. Cytotechnicians and supervising pathologists were blind to the women’s HPV status.

### Diagnostic confirmation

Colposcopy with directed biopsy was considered the gold standard for diagnostic confirmation. These procedures were performed by specialists at the health centers’ referral hospital, as part of their regular activities; according to standardized protocol, biopsies were taken of all areas with acetowhite lesions or atypical vessels. Histological examination of the biopsies was performed by hospital pathologists, with regular internal quality control procedures in place; results were reported according to standard cervical intraepithelial neoplasia (CIN) terminology. Women with negative colposcopies were considered without lesions.

### Analysis

Diagnostic accuracy of HPV16/18 genotyping for the detection of CIN2+ and CIN3+ among hrHPV positive women was assessed calculating sensitivity, specificity, positive predictive value (PPV) and negative predictive value (NPV), with 95 % confidence intervals (CI); crude estimates were obtained based on women with complete screening, triage and diagnostic confirmation results.

## Abbreviations

ASCUS: Atypical squamous cells of undetermined significance
ASCUS+: ASCUS or worse
CI: Confidence interval
CIN: Cervical intraepithelial neoplasia
CIN2 +: CIN grade 2 or worse
CIN3 +: CIN grade 3 or worse
HC2: Hybrid Capture 2
HPV: Human papillomavirus
hrHPV: High-risk HPV
Pap: Papanicolaou
PPV: Positive predictive value
NPV: Negative predictive value
